# Inhibition of interleukin‐12 and/or interleukin‐23 for the treatment of psoriasis: What is the evidence for an effect on malignancy?

**DOI:** 10.1111/exd.13676

**Published:** 2018-06-28

**Authors:** Elizabeth N. Ergen, Nabiha Yusuf

**Affiliations:** ^1^ Department of Dermatology University of Alabama at Birmingham Birmingham AL USA

**Keywords:** biological therapy, cancer, interleukin, psoriasis

## Abstract

Immune cells and cytokines play an important role in the pathogenesis of psoriasis. Interleukin‐12 (IL‐12) and IL‐23 promote cellular responses mediated by T cells, which contribute to an inflammatory loop responsible for the induction and maintenance of psoriatic plaques. Antibodies that inhibit IL‐12/23 or IL‐23 are key treatment options for patients with psoriasis. IL‐12 and IL‐23 also play a key role in immune responses to infections and tumors. A growing body of information from clinical trials, cohort studies, postmarketing reports, genetic studies and animal models provides insights into the potential biological relationships between IL‐12/23 inhibition and malignancies. We summarize this information in tables and provide some context for the interpretation of these data with the goal of informing dermatologists who are using IL‐12/23 or IL‐23 inhibitors to treat patients with psoriasis.

## 1 INTRODUCTION

**Figure 1:**
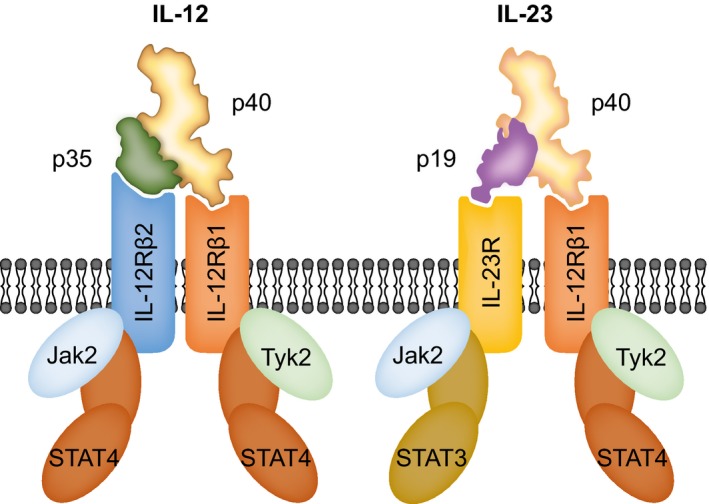
Structure of IL‐12 and IL‐23 cytokines and receptors. IL, interleukin; Jak, Janus kinase; R, receptor; STAT, signal transducers and activators of transcription; Tyk, tyrosine kinase

Psoriasis is a chronic immune‐mediated disease that affects 3.2% of the adult US population^1^ and is characterized by scaly red plaques^2^ associated with a diminished quality of life.^2^, ^3^ Psoriasis is associated with multiple comorbidities, including arthritis, diabetes, cardiovascular disease and depression,^4^, ^5^ as well as decreased life expectancy in patients with severe disease.^6^ An increased risk of cancer has been observed among patients with psoriasis compared with the general population.^7^ Cohort studies have shown that patients with psoriasis have an increased risk of specific cancers regardless of their treatment, including non‐melanoma skin cancers (NMSCs), lymphohematopoietic cancers and cancers of the respiratory and digestive tracts.^7^, ^8^, ^9^ The interleukin‐12 (IL‐12)/23 inhibitor ustekinumab is an established treatment for psoriasis, and IL‐23–specific inhibitors are among the newest therapies, with promising efficacy and safety profiles (Table 1; Figure 1).^10^, ^11^ IL‐12 and IL‐23 play a central role in T‐cell–mediated immunity and the proinflammatory responses associated with autoimmune conditions.^12^, ^13^, ^14^, ^15^, ^16^, ^17^, ^18^ However, in vitro and animal studies have suggested that IL‐12 and IL‐23 may have distinct roles in contributing to protective immune responses to tumors^19^, ^20^, ^21^ and bacterial infections.^12^, ^22^ Thus, therapies targeted to IL‐12 and IL‐23 carry a theoretical risk of decreased defenses against pathogens and tumor surveillance. A concern for an increased risk of malignancy has been raised with other immunosuppressive therapies used for the treatment of psoriasis, such as anti–tumor necrosis factor (TNF) inhibitors, owing to the role of TNF in tumor growth inhibition.^23^ This is a review of the currently available information on the role of IL‐12 and IL‐23 in tumor growth and is written for clinicians who want to understand the potential risk of malignancy associated with blocking IL‐12 and/or IL‐23 in the treatment of psoriasis.

**Table 1:** Inhibitors of IL‐12/23 or IL‐23 licensed or in clinical development for the treatment of psoriasis

Generic name [compound] (brand name)	Antibody type	Mechanism of action	Manufacturer	References
Ustekinumab [CNTO‐1275] (Stelara^®^)	Fully human IgG1κ monoclonal antibody	Binds with high affinity to IL‐12/23p40 subunit	Janssen Biotech Inc.	Kauffman et al^85^
Briakinumab^a^ [ABT‐874, J‐695] (Ozespa)	Fully human IgG1λ monoclonal antibody	Binds to IL‐12/23p40 subunit	Abbott Laboratories Ltd	Fragoulis et al,^86^ Panaccione et al^87^
Guselkumab [CNTO 1959] (Tremfya^™^)	Fully human IgG1λ monoclonal antibody	Binds to IL‐23p19 subunit	Janssen Biotech Inc.	Reich et al,^11^ Gordon et al^88^
Tildrakizumab [MK‐3222, SCH 900222] (Ilumya^™^)	Humanized mouse IgG1κ monoclonal antibody	Binds with high affinity to IL‐23p19 subunit (297 pmol/L)	Merck & Co, Inc., and Sun Pharmaceutical Industries, Inc.	Reich et al,^11^ Papp et al^89^
Risankizumab [ABBV‐066, BI 655066]	Humanized IgG1κ monoclonal antibody	Binds with high affinity to IL‐23p19 subunit (dissociation constant <10 pmol/L)	Boehringer Ingelheim and AbbVie Inc.	Krueger et al,^90^ Papp et al,^39^ Singh et al^91^
Mirikizumab [LY3074828]	Humanized monoclonal antibody	Blocks IL‐23	Eli Lilly and Company	Eli Lilly and Company^92^

Ig, immunoglobulin; IL, interleukin.

a US and European license applications withdrawn by manufacturer in 2011.^37^

## 2 STRUCTURE AND BIOLOGICAL EFFECTS OF IL‐12 AND IL‐23 IN PSORIASIS

IL‐12 and IL‐23 are heterodimers, sharing a common p40 (beta chain) subunit that is combined with either a p35 alpha chain (IL‐12) or p19 alpha chain (IL‐23; Figure 1).^13^, ^18^ IL‐12 and IL‐23 signal through heterodimeric receptors, both of which contain IL‐12 receptor β1 (IL‐12Rβ1), which is coupled with IL‐12Rβ2 to form the IL‐12 receptor and with IL‐23R to form the IL‐23 receptor.^17^, ^18^ Signalling, mediated through the Janus kinase–signal transducers and activators of transcription (Jak‐STAT) pathway, ultimately results in IL‐12 and IL‐23 promoting the development of cell‐mediated responses driven by different subsets of T helper (T_H_) cells.^17^, ^18^

**Figure 2:**
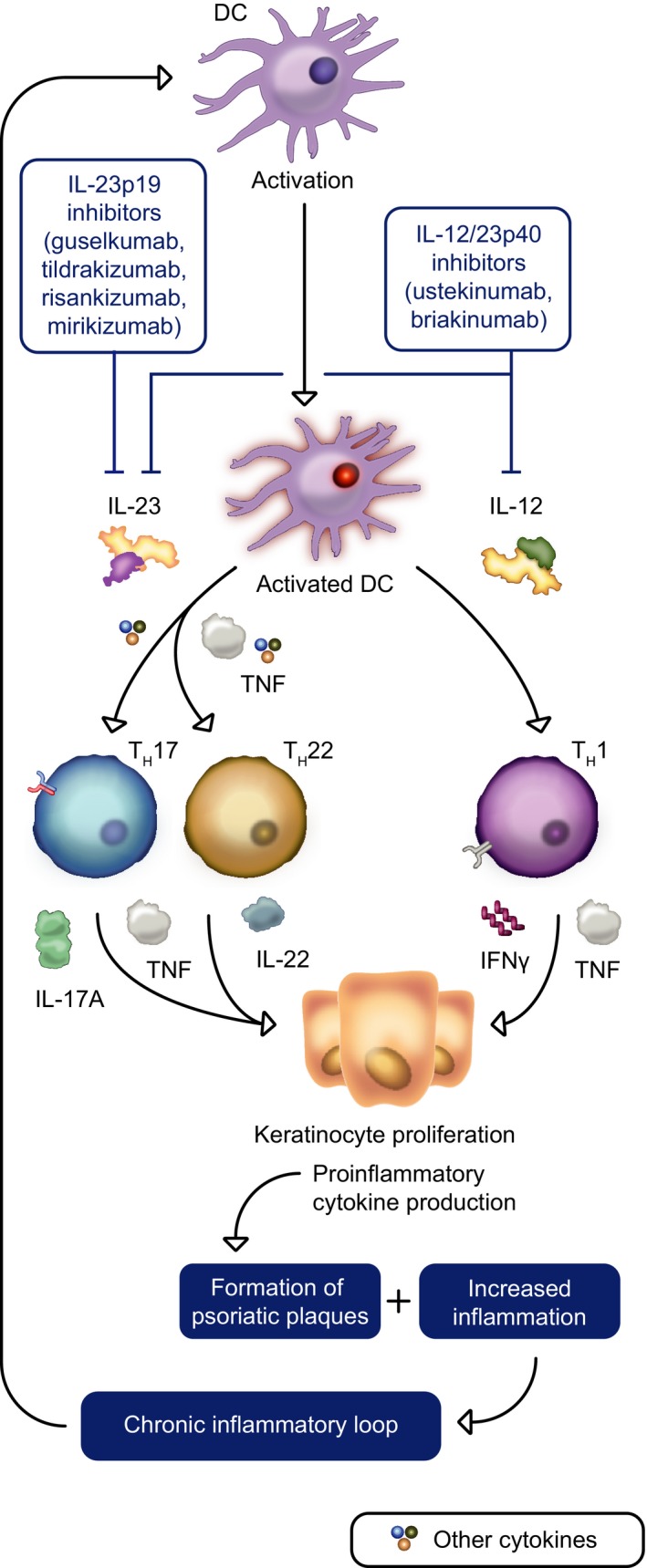
Biological effects of IL‐12 and IL‐23 and their inhibitors in psoriasis. DC, dendritic cell; IFN, interferon; IL, interleukin; T_H_, T helper; TNF, tumor necrosis factor

IL‐12 plays a key role in differentiation, maintenance and activity of immune cell subsets, including T_H_1 cells (which produce interferon‐γ) and natural killer cells (Figure 2).^12^, ^16^, ^24^ IL‐23 has a key role in maintenance and activity of IL‐17–producing T_H_17 cells and IL‐22–producing T_H_22 cells.^25^ In turn, IL‐17 induces activation and proliferation of keratinocytes, which produce inflammatory cytokines, including IL‐23, leading to a self‐amplificatory loop.^26^, ^27^ Consequently, antibodies targeted to IL‐12 and IL‐23 (p40 inhibitors) affect T_H_1, T_H_17 and T_H_22 responses, whereas those targeted to IL‐23 alone (p19 inhibitors) primarily affect T_H_17 and T_H_22 responses.^26^, ^27^

## 3 INHIBITORS OF IL‐12/23 or IL‐23 FOR THE TREATMENT OF PSORIASIS

The development of anti–IL‐12/23 antibodies was originally initiated based on the observation that mice deficient in the IL‐12p40 subunit (the subunit shared by IL‐12 and IL‐23) and those treated with neutralizing anti–IL‐12p40 antibodies showed resistance to autoimmunity.^15^ The discovery of IL‐23^18^ followed by the molecular characterization of IL‐23^17^ and other murine and genetic studies helped identify the key pathogenic role of IL‐23 in psoriasis, as summarized by Gaffen et al.^25^ It is now understood that IL‐12 and IL‐23 act on different components of the chronic inflammatory loop associated with the formation of psoriatic plaques.^28^ An association between IL‐12 and psoriasis is supported by the presence of T_H_1 cells and interferon‐γ in psoriatic lesions.^29^ However, recent data from a mouse model of psoriasis suggest that IL‐12 may dampen skin inflammation in psoriasis by modulating IL‐23–mediated inflammatory events, decreasing skin invasion by T_H_17 cells and promoting an anti‐inflammatory genetic programme in keratinocytes.^30^

Two antibodies targeting IL‐12/23p40—ustekinumab and briakinumab—have been evaluated as treatments for psoriasis and other immune‐mediated diseases.^31^, ^32^, ^33^, ^34^, ^35^ Ustekinumab is the only IL‐12/23p40 inhibitor for the treatment of moderate‐to‐severe plaque psoriasis and psoriatic arthritis approved by the Food and Drug Administration (FDA).^36^ Clinical development of briakinumab was discontinued, thought to be because of safety concerns reported in the clinical trials, including cardiac events and malignancies.^31^, ^37^ Guselkumab was the first antibody specifically targeting IL‐23p19 to be approved for the treatment of moderate‐to‐severe psoriasis^38^, and 3 further IL‐23p19 inhibitors are currently in active development for the same indication. Efficacy and safety data have been published for tildrakizumab (phase 3^11^) and risankizumab (phase 2^39^); mirikizumab (LY3074828) is currently entering phase 2 development.^40^

## 4 MALIGNANCIES REPORTED IN CLINICAL TRIALS

A variety of cancer types have been reported in clinical trials of IL‐12/23 and IL‐23 inhibitors (Table 2). NMSCs were the most frequently reported malignancies. These are the most common malignancies in humans (albeit not routinely reported to cancer registries), with basal cell carcinomas (BCCs) more common than squamous cell carcinomas (SCCs).^41^ A meta‐analysis of observational studies found that the risk of SCCs was increased in patients with psoriasis compared with the general population (standardized incidence ratio [SIR] = 5.31, 95% confidence interval [CI] = 2.63–10.71) and correlated with patient exposure to 8‐methoxypsoralen–ultraviolet (UV) A therapy for treatment of psoriasis.^9^ Risk of BCCs was also increased in patients with psoriasis, but to a lesser extent than SCCs (SIR = 2.00, 95% CI = 1.83–2.20).^9^ In trials of ustekinumab for psoriasis, as in the general population,^41^ the proportion of BCCs was higher than that of SCCs.^32^, ^42^, ^43^, ^44^ However, a pooled analysis of safety data from all briakinumab phase 2 and phase 3 trials and interim data from an open‐label extension trial suggested that the risk of SCC was similar to the risk of BCC in patients treated with briakinumab, which may suggest a relative or absolute increase in the risk of SCC.^45^ Concern about the effect of briakinumab on NMSCs was thought to be one of the reasons for discontinuing its development.^31^, ^37^

**Table 2:** Reported malignancies in clinical trials of IL‐12/23 and IL‐23 inhibitors in patients with moderate‐to‐severe psoriasis receiving active treatment with an IL‐12/23 OR IL‐23 inhibitor

Inhibitor	Phase	Name	NCT #	Study length	N	Treatment arms	Cases of reported malignancies					Reference
							BCC	SCC	Prostate	Breast	Others	
Ustekinumab	1	Single dose	–	16 wk	18	0.1–5 mg/kg					None	Kauffman et al^85^
	2	–	00320216	36 wk	320	45 or 90 mg^a^	2	1	1		None	Krueger et al^44^
	3	PHOENIX 1	00267969	76 wk	766	45 or 90 mg^a^	4		1	1	4: thyroid cancer, lentigo maligna, colon cancer, transitional cell carcinoma	Leonardi et al^32^
	Ext	PHOENIX 1 OLE	–	>5 y (264 wk)	753	45 or 90 mg	13	1	5	1	9: 3 melanomas (2 in situ, 1 invasive), and 1 each of colon cancer, lymphoma, metastatic pancreatic carcinoma, head/neck cancer, thyroid cancer, transitional cell carcinoma	Kimball et al^43^
	3	PHOENIX 2	00307437	52 wk	1230	45 or 90 mg^a^	7^b^				2: hepatocellular carcinoma, SCC of the tongue	Papp et al^33^
	3	ACCEPT	00454584	64 wk	903	45 or 90 mg^c^	6 + 2^d^	1 + 2^d^	1	1	3: oral neoplasm, chronic lymphocytic leukaemia, mycosis fungoides	Griffiths et al^42^
	2^e^	Dose ranging	02054481	48 wk	166	45 or 90 mg					None	Papp et al^39^
	3^f^	NAVIGATE	02203032	60 wk	871	45 or 90 mg	3	1			2: bile duct cancer, pancreatic carcinoma	Langley et al^93^
Briakinumab	2	M05‐736	00292396	12 wk	180	100 or 200 mg^a^		1			None	Kimball et al^94^
	3	M10‐114	00691964	12 wk	347	200–100 mg^a^^,^ c					1 malignant melanoma in situ	Gottlieb et al^95^
	3	M10‐315	00710580	12 wk	350	200–100 mg^a^^,^ c	1				2: colon cancer, lip neoplasm	Strober et al^96^
	3	M10‐255	00679731	52 wk	317	200–100 mg^g^		1	1	1	2: transitional cell carcinoma, breast neoplasm‐intraductal carcinoma	Reich et al^97^
	3	M06‐890	00570986	52 wk	1465	200–100 mg^a^	4	6			4: nasopharyngeal, tonsillar, lung, and colon cancers	Gordon et al^31^
Guselkumab	1	Single, ascending dose	00925574	24 wk	24	10–300 mg^a^					None	Sofen et al^98^
	2	X‐PLORE	01483599	52 wk	293	5–200 mg^h^					1 cervical intraepithelial neoplasia	Gordon et al^88^
	3	VOYAGE 1	02207231	48 wk	837	100 mg^h^	2		1	1		Blauvelt et al^46^
	3	VOYAGE 2	02207244	72 wk	992	100 mg^h^	2	2	1			Reich et al^10^
	3	NAVIGATE	02203032	60 wk	871	100 mg^i^		1			1 transitional cell carcinoma of the bladder	Langley et al^93^
Tildrakizumab	1	Sequential, rising, multiple dose	–	16 wk	77	0.05–10 mg/kg^a^					None	Kopp et al^99^
	2b	Dose finding	01225731	52 wk	353	5–200 mg^a^					1 malignant melanoma	Papp et al^89^
	3	reSURFACE 1	01722331	64 wk^i^	772	100 mg, or 200 mg^a^	3^b^				4: unspecified	Reich et al^11^
	3	reSURFACE 2	01729754	52 wk^i^	1090	100 mg, or 200 mg^a^^,^ c	4^b^				4: unspecified	Reich et al^11^
Risankizumab	1	Single, rising dose	01577550	24 wk	39	0.01–5 mg^a^					None reported	Krueger et al^90^
	2	Dose ranging	02054481	48 wk	166	18, 90, or 180 mg^j^	2				1 salivary gland neoplasm	Papp et al^39^

BCC, basal cell carcinoma; IL, interleukin; NCT, national clinical trial; NMSC, non‐melanoma skin cancer; OLE, open‐label extension; SCC, squamous cell carcinoma.

Blank cells indicate no cases were reported in the publication.

a vs placebo.

b BCC + SCC combined.

c vs etanercept.

d 2 cases had both BCC and SCC.

e Active control vs risankizumab.

f Active control vs guselkumab.

g vs methotrexate.

h vs placebo or adalimumab.

i Data are reported up to week 28.

j vs ustekinumab.

The clinical evaluation of IL‐23p19 inhibitors is ongoing, and data available thus far are limited. NMSCs have been reported in some clinical trials.^10^, ^11^, ^39^, ^46^ Publications about tildrakizumab reported low numbers (7 cases total) of NMSCs but did not differentiate BCCs and SCCs.^11^ Two cases of BCCs were reported in the phase 2 trial of risankizumab.^39^

Prostate and breast cancers are the most common internal malignancies in men and women, respectively.^47^ Patients with psoriasis have not been found to have a significantly increased risk of prostate cancer compared with the general population,^7^, ^8^ but the relative risk of breast cancer in patients with psoriasis is less clear. Two analyses showed no significant increased risk,^7^, ^8^ and one showed a slightly increased risk compared with the general population.^9^ Several cases of prostate and breast cancers occurred in trials of ustekinumab,^32^, ^42^, ^43^, ^44^ briakinumab^34^ and guselkumab.^10^, ^46^ These malignancy events reported in clinical trials do not prove causation but do suggest a possible biological relationship that may trigger further investigation.^48^

## 5 MALIGNANCIES REPORTED IN POSTMARKETING SAFETY DATA

Ustekinumab, which has been approved since 2009, is currently the only IL‐12/23 or IL‐12 inhibitor with postmarketing safety data. The prescribing information (PI) for ustekinumab contains a general warning that it “may increase risk of malignancy,” based on the observations that (i) NMSCs were reported in 1.5% of patients and malignancies excluding NMSCs (non‐NMSCs) were reported in 1.7% of patients among patients treated with ustekinumab (3.2 years’ median follow‐up); (ii) the most frequently observed non‐NMSCs were prostate, melanoma, colorectal and breast cancers, but they were similar in type and number to those expected in the general US population when adjusted for age, gender and race; and (iii) rapid appearance of multiple cutaneous SCCs was found in postmarketing reports among patients receiving ustekinumab who had pre‐existing risk factors for developing NMSC.^36^ The concerns raised in the ustekinumab PI are in line with an analysis of postmarketing safety data reported to the FDA, which found that patients treated with ustekinumab were 15 times more likely to report a case of cancer than were patients treated with apremilast, a phosphodiesterase 4 (PDE‐4) inhibitor.^49^ Furthermore, a safety signal was detected in a study of data from the FDA Adverse Event Reporting System database, which indicated that ustekinumab may be associated with several malignancies, including B‐cell lymphoma; epithelioid sarcoma; and lung, oesophageal, ovarian, renal, testis and thyroid cancers.^50^

In contrast, an analysis of data from the Psoriasis Longitudinal Assessment and Registry (PSOLAR) showed that patients with psoriasis treated with ustekinumab had numerically fewer non‐NMSCs (0.48/100 patient‐years [PYs]) than did patients treated with infliximab, a monoclonal antibody directed at TNF (0.79/100 PYs) or any other biologics (0.73/100 PYs) or non‐biologics (0.84/100 PYs).^51^ Results from an analysis of the German Psoriasis Registry (PsoBest) showed similar rates of malignancies excluding NMSCs in patients receiving systemic therapies (0.46/100 PYs) or biologics (0.49/100 PYs), with no relevant differences between therapies.^52^ Evidence from controlled clinical trials and registries indicates that ustekinumab is well tolerated, with rates of overall mortality and malignancy comparable with those expected in the general population.^32^, ^33^, ^53^, ^54^ Although the postmarketing pharmacovigilance studies mentioned previously were not designed to study causality or to quantify increased cancer risk associated with specific therapies,^49^, ^50^ they can be helpful to identify safety signals that may be relevant to the advancement of overall patient safety.^55^ Postmarketing safety data are not yet available for tildrakizumab, guselkumab or risankizumab. Long‐term data are needed to identify any potential associations between IL‐23 inhibitors and malignancies.

Clinical trials detect adverse events during trials of relatively short duration. For example, the trials of IL‐12/23 and IL–23 inhibitors summarized in Table 2 varied in duration from 12 to 76 weeks. Consequently, drugs are usually made available for public use before rare but potentially serious adverse reactions have been identified and their probabilities quantified.^48^ Although NMSCs may develop within weeks to months^56^ and are relatively easy to detect by visual inspection, other malignancies may take longer to be discovered. Thus, the incidence of internal malignancy, and even NMSCs, in a clinical trial may not be a good indicator of a drug's long‐term effect on malignancy. Malignancies that occur infrequently or develop slowly may only be detected in postmarketing evaluations, making them a critical element of drug safety monitoring efforts. An effect on pre‐existing malignancies is difficult to assess as most trials exclude these patients from receiving systemic immunosuppressive drugs. For example, trials for ustekinumab, briakinumab, guselkumab, tildrakizumab and risankizumab all excluded patients with a pre‐existing malignancy in the preceding 5 years (except fully treated BCCs or SCCs of the skin and/or fully treated cervical carcinoma in situ).^10^, ^11^, ^31^, ^32^, ^39^, ^46^

## 6 RISK OF MALIGNANCY IN HUMANS WITH GENETIC TRAITS SIMULATING NEUTRALIZATION OF IL‐12 AND/OR IL‐23

In the first report of cancer in a patient with IL‐12Rβ1 deficiency (the receptor subunit required to bind the p40 subunit shared by IL‐12 and IL‐23), the patient developed oesophageal SCC at age 25 years and died at age 29 years, an age at which this cancer is exceedingly rare (Table 3).^57^ Additional studies of IL‐12 genetic deficiencies have investigated whether such deficiencies are associated with increased likelihood of infections and cancer.^58^, ^59^ Genomewide association studies have shown that polymorphisms in genes encoding the IL‐12p40 subunit or the IL‐12p35 subunit, which result in a decreased biological effect of IL‐12, are linked to increased susceptibility to oesophageal cancer,^60^ osteosarcoma,^61^ bladder cancer^62^ and prostate cancer,^63^ as well as susceptibility to and/or severity of cervical cancer.^64^ Reports of genetic deficiencies simulating a deficiency in the IL‐23–signalling pathway are limited. Several genomewide association studies have been conducted in Chinese populations; they show that variants of *IL‐23R*, the subunit specific for the IL‐23 receptor, are associated with a significantly reduced risk of gastric cancer,^65^ but with a significantly increased risk of oesophageal cancer,^66^ hepatocellular carcinoma^67^ and acute myeloid leukaemia.^68^ However, the effect of these *IL‐23R* variants on the function of IL‐23 (eg, gain, loss or no effect) was not specifically described in the studies. Taken together, these findings might lead to the hypothesis that IL‐12/23 inhibitors have the potential to increase the risk of these cancers. However, a limitation of genomewide association studies is that they are not designed to investigate the causal relationship between a specific polymorphism and an increased cancer risk. For example, although several studies had shown that the TNF‐238 polymorphism increased cancer risk, a meta‐analysis of 34 studies did not find a significant association between this polymorphism and increased cancer risk.^69^

**Table 3:** IL‐12/23 and IL‐23 genetic deficiencies associated with increased risk of cancer

Gene mutation or polymorphism	Effect on IL‐12 and/or IL‐23	Effect on malignancy	Potential implications for therapy with IL‐12/23 or IL‐23 inhibitors
*IL‐12R*β*1* homozygous deficiency case report	Loss of IL‐12 and IL‐23 functions	Oesophageal squamous cell carcinoma at age 25 y, relapse and death at age 29 y^57^	IL‐12/23 inhibitors may increase risk of oesophageal cancer
*IL‐12R*β*1* polymorphism: 378 GG/GC vs CC	Decreased IL‐12 levels	Increased risk of oesophageal cancer^60^	IL‐12/23 inhibitors may increase risk of oesophageal cancer
*IL‐12B* (IL‐12p40) polymorphisms: rs321227 AC/CC or CC vs AA, or C vs A	Decreased IL‐12 levels	Increased risk of osteosarcoma^61^ and oesophageal^60^ and prostate^63^ cancers	IL‐12/23 inhibitors may increase risk of osteosarcomas and bladder, cervical, oesophageal and prostate cancers
1188 AC vs AA		Increased risk of bladder cancer^62^	
rs2569254 GG vs AA		Increased risk of cervical cancer^64^	
*IL‐12A* (IL‐12p35) polymorphisms: rs568408 GA/AA or GA vs GG	Decreased IL‐12 levels	Increased risk of oesophageal cancer^60^ and osteosarcoma^61^	IL‐12/23 inhibitors may increase risk of osteosarcomas and oesophageal cancer in specific patient populations
*IL‐23R* polymorphisms: rs6682925 TC/CC or TG/GG or T>C( )rs1884444n T>G	Loss of IL‐23 function	Increased risk of oesophageal cancer,^66^ hepatocellular carcinoma,^67^ and acute myeloid leukaemia^68^ No association with risk of gastric cancer^65^ Decreased risk of gastric cancer^65^	IL‐23 inhibitors may affect cancer risk of some cancers in specific patient populations IL‐23 inhibitors may decrease risk of gastric cancer

IL, interleukin; R, receptor.

## 7 RISK OF MALIGNANCY IN ANIMAL MODELS SIMULATING NEUTRALIZATION OF IL‐12 AND/OR IL‐23

The malignancy data from animal models of IL‐23 deficiency are conflicting. Mice that had lost IL‐23 function via deficiencies in either *IL‐23p19* or *IL‐23R* or by treatment with antibodies to IL‐23p19 showed resistance to skin tumor growth/development (Table 4).^20^ IL‐23–deficient mice^70^ and mice treated with anti–IL‐23p19^71^ have also been shown to have an increased resistance to melanoma‐induced lung metastases. Furthermore, in this model of melanoma‐induced metastases, anti–IL‐23 antibody used in combination with IL‐2 or anti‐erbB2 antibody significantly inhibited subcutaneous growth of established mammary carcinomas and suppressed established and spontaneous lung metastases.^71^ Deficiencies in *IL‐23p19* or *IL‐23R* also resulted in decreased tumor multiplicity and growth in a mouse model of colorectal tumors.^72^ These findings suggest that IL‐23p19 inhibitors might prevent the growth and/or enhance the rejection of some tumors, possibly via effects on IL‐22, which has been implicated in the development of epithelial tumors.^73^, ^74^ A number of studies have found that increased levels of IL‐23 are associated with unfavourable outcomes in various malignancies in humans.^75^, ^76^, ^77^, ^78^, ^79^ In contrast, other studies suggest that IL‐23p19 deficiency might enhance the risk of certain cancers. For example, IL‐23–deficient mice demonstrated an increased risk of development of chemically induced melanoma.^80^ However, in a model of UV radiation, IL‐23–deficient mice demonstrated both an increased risk of developing sarcoma and a decreased risk of developing epithelial tumors compared with wild‐type mice.^19^ Further studies are needed to confirm this finding.

**Table 4:** Malignancies in murine models of IL‐23 deficiency

Model	Effect on IL‐23	Tumor‐promotion strategy	Effect on malignancy vs controls	Potential therapeutic implications for IL‐23 inhibitors
Treatment with anti–IL‐23p19 antibody^20^	Loss of IL‐23 function	Intradermal injection of skin tumor cells	Faster rejection of tumor cells and decreased tumor formation	May prevent tumor growth and enhance tumor rejection
Treatment with anti–IL‐23p19 antibody^71^	Loss of IL‐23 function	Experimental and spontaneous models of lung metastases SC injection of thymoma cells	Early suppression of lung metastases and modest inhibition of primary tumors with subcutaneous growth	May prevent tumor growth and metastasis
*IL‐23p19* ^−/−[^ 20 ^]^	Loss of IL‐23 function	Chemical carcinogenesis Intradermal injection of skin tumor cells	Resistance to developing skin papillomas Resistance to developing tumors	May reduce risk of skin cancer May prevent tumor growth and enhance tumor rejection
*IL‐23p19* ^−/−[^ 70 ^]^	Loss of IL‐23 function	Experimental model of lung metastases	Increased resistance to formation of lung metastases	May prevent tumor growth and enhance tumor rejection
*IL‐23p19* ^−/−[^ 72 ^]^	Loss of IL‐23 function	Colorectal tumorigenesis in genetically predisposed mice	Decreased tumor number and growth	May prevent tumor growth and enhance tumor rejection
*IL‐23p19* ^−/−[^ 19 ^]^	Loss of IL‐23 function	Skin UV radiation	Increased probability of skin tumor development	May increase risk of UV radiation–induced skin cancer
*IL‐23p19* ^−/−[^ 80 ^]^	Loss of IL‐23 function	Chemically induced melanoma Chemically induced epithelial tumor	Increased number and size of melanomas Resistance to tumor development	May increase risk of melanoma May decrease risk of epithelial tumors
*IL‐23R* ^−/−[^ 20 ^]^	Loss of IL‐23 receptor function	Intradermal injection of tumor cells	Resistance to tumor development	May prevent tumor growth and enhance tumor rejection
*IL‐23R* ^−/−[^ 72 ^]^	Loss of IL‐23 receptor function	Colorectal tumorigenesis in genetically predisposed mice	Decreased tumor number and growth	May prevent tumor growth and enhance tumor rejection

IL, interleukin; SC, subcutaneous; UV, ultraviolet.

Similarly, the studies on IL‐12 also show conflicting data. Several studies of mice with IL‐12–specific loss of function via deficiency in *IL‐12p35* 20 or *IL‐12R*β*2* 81 showed an increased risk of tumor development (Table 5), but, in models of UV radiation^19^ or chemically induced melanomas,^80^ *IL‐12p35*–deficient mice had the same risk of induced skin tumors as did their wild‐type counterparts.

**Table 5:** Malignancies in murine models of IL‐12 deficiency

Model	Effect on IL‐12	Tumor‐promotion strategy	Effect on malignancy vs controls	Potential therapeutic implications for IL‐12 inhibitors
*IL‐12p35* ^−/−[^ 83 ^]^	Loss of IL‐12 function	Spontaneous tumor development	No effect	No impact on malignancy
*IL‐12p35* ^−/−[^ 20 ^]^	Loss of IL‐12 function	Chemical carcinogenesis Intradermal injection of skin tumor cells	Earlier and more frequent skin papillomas Increased incidence of skin tumors	May increase risk of skin cancer
*IL‐23p35* ^−/−[^ 70 ^]^	Loss of IL‐12 function	Experimental model of lung metastases	Increased formation of lung metastases	May increase growth and reduce tumor rejection
*IL‐12p35* ^−/−[^ 100 ^]^	Loss of IL‐12 function	Skin UV radiation	Increased number of skin tumors	May increase risk of skin cancer
*IL‐12p35* ^−/−[^ 19 ^]^	Loss of IL‐12 function	Skin UV radiation	No increased probability of skin tumor development	May not affect risk of UV radiation–induced skin cancer
*IL‐12p35* ^−/−[^ 80 ^]^	Loss of IL‐12 function	Chemically induced melanomas Chemically induced epithelial tumor	Reduced number and size of melanomas Increased tumor development	May reduce risk of melanoma May increase risk of epithelial tumors
*IL‐12R*β*2* ^−/−[^ 81 ^]^	Loss of IL‐12 receptor function	Spontaneous	Increased susceptibility to spontaneous tumor formation, half of aged mice developed plasmacytoma or lung epithelial tumors	May increase risk of cancer

IL, interleukin; UV, ultraviolet.

Mouse models with loss of function in both IL‐12 and IL‐23 via IL‐12/23p40 deficiency or treatment with anti–IL‐12/23p40 antibodies showed an increase,^20^, ^21^, ^80^ a decrease^82^ or no difference^83^ in tumor development (Table 6).

**Table 6:** Malignancies in murine models of IL‐12/23p40 deficiency

Model	Effect on IL‐12/23	Tumor‐promotion strategy	Effect on malignancy vs controls	Potential therapeutic implications for IL‐12/23p40 inhibitors
Treatment with anti–IL‐12/23p40 antibodies^20^	Loss of IL‐12 and IL‐23 function	Intradermal injection of skin tumor cells	Increased number and size of faster‐growing tumors	May increase risk of cancer, tumor growth, and metastases
Treatment with anti–IL‐12p40 antibody^71^	Loss of IL‐12 and IL‐23 function	Experimental and spontaneous models of lung metastases	No effect	No impact on malignancy
*IL‐12/23p40* ^−/−[^ 20 ^]^	Loss of IL‐12 and IL‐23 function	Chemical carcinogenesis Intradermal injection of skin tumor cells	Resistance to developing skin papillomas Increased tumor incidence	May reduce risk of skin cancer May increase risk of skin cancer
*IL‐23p40* ^−/−[^ 70 ^]^	Loss of IL‐12 and IL‐23 function	Experimental model of lung metastases	No effect	No impact on malignancy
*IL‐12/23p40* ^−/−[^ 21 ^]^	Loss of IL‐12 and IL‐23 function	Skin UV radiation	Increased skin tumor development	May increase risk of UV radiation–induced skin carcinogenesis
*IL‐12/23p40* ^−/−[^ 82 ^]^	Loss of IL‐12 and IL‐23 function	Chemically induced skin tumors	Resistance to skin tumor development	May reduce risk of skin cancer
*IL‐12/23p40* ^−/−[^ 80 ^]^	Loss of IL‐12 and IL‐23 function	Chemically induced melanomas Chemically induced epithelial tumor	Increased number and size of melanomas Increased number of epithelial tumors	May increase risk of melanoma May increase risk of epithelial tumors

IL, interleukin; UV, ultraviolet.

## 8 CONCLUSIONS

Patients with psoriasis and/or receiving treatment for psoriasis have an increased risk of cancer.^8^, ^9^, ^49^, ^84^ Inhibitors of IL‐12/23 and IL‐23 are effective treatment approaches for psoriasis.^36^, ^38^ Existing data provide evidence to support an association between impaired IL‐12 and/or IL‐23 signalling and both tumor growth and resistance to tumor growth, although the nature of these relationships is not fully understood. Long‐term postmarketing safety evaluations of agents targeting IL‐12/23 and IL‐23 are needed to fully appreciate the associated malignancy risk. The implications for therapeutic inhibition of IL‐12/23 or IL‐23 remain uncertain, although monitoring of patients for NMSC and malignancy seems warranted.

## CONFLICT OF INTEREST

The authors have declared no conflicting interests.
